# Supplementary material for: Individuality across environmental context in Drosophila melanogaster
Source: eLife. 2026 Apr 13;13:RP98171. doi: 10.7554/eLife.98171 (PMC13075937; doi:10.7554/eLife.98171)
Supplement: Supplementary file 1. [file elife-98171-supp1.pdf]

Parameter	Description
roinr	Arena number for fly identification.
sex	Male = 0, female = 1.
eucldistsum	Total distance walked in mm.
perctimemoving	% of time spent walking.
numberpauses	Number of pauses (min pause length = 1s).
medianpausedurationsec	Median pause duration in s.
meanspeedmov	Mean walking speed in mm/s.
mediananglecentermoving	Median walking angle (heading direction) between 0° and 360° in the center region of the arena as seen from the animal.
mediananglecenteraxialmoving	Median walking angle (heading direction) between 0° and 180° (axial) in the center region of the arena as seen from the animal. Axial transformation of angular data was achieved by subtracting 180 ° from all angles larger than 180°. Axial data is more robust against cancelling -out of opposing angles in median calculations, e.g. when a fly is walking back and forth between stripes.
stddeviation	Standard deviation of angular data in ° as seen from the animal. Low angular standard deviation indicates a smaller range of chosen heading directions. Contains only data points where the animal was walking in the center region of the arena.
rlength	Resulting vector length of angular data mapped between 0° and 360°. A vector length of 0 would indicate uniform distribution of heading angles. A vector length of 1 would indicate the animl was only moving in a perfectly straight path. Contains only data points where the animal was walking in the center region of the arena.
rlengthax	Resulting vector length of angular data mapped between between 0° and 180° (axial) . A vector length of 0 would indicate uniform distribution of heading angles. A vector length of 1 would indicate the animl was only moving in a perfectly straight path. Contains only dat a points where the animal was walking in the center region of the arena. Axial data is more robust against cancelling -out of opposing angles in median calculations, e.g. when a fly is walking back and forth between stripes.
medianangvelmovalldegs	Median angular velocity (angular speed of azimuthal rotation) when walking in °/s. Median angular velocity is also a measure of handedness. Positive values indiceate right turns and negative values indicate left turns as seen from the animal.
medianangvelmovcenterdegs	Median angular velocity (angular speed of azimuthal rotation) when walking in the center region of the arena in °/s. Median angular velocity is also a measure of handedness. Positive values indiceate right turns and negative values indicate left turns as seen from the animal.
medianangvelmovouterdegs	Median angular velocity (angular speed of azimuthal rotation) when walking in the edge region of the arena in °/s. Median angular velocity is also a measure of handedness. Positive values indiceate right turns and negative values indicate left turns as seen from the animal.
absturninganglesum	Sum of all walking heading changes in °. Divided by distance walked this can be used as a measure for „meandering“ (heading change per mm walked in °/mm).
absturninganglesumcenter	Sum of all walking heading changes within the center region of the arena in °. Divided by distance walked this can be used as a measure for „meandering“ (heading change per mm walked in °/mm).
centrophobindex	Centrophobicity index between 0 and 1. 0 indicates an animal being in the center area 100% of the time. 1 indicates an animal being in the edge area 100% of the time.
pecflymorethanone	% of time with more than two objects detected per arena. This parameter indicates false animal detections (e.g dust) and and is useful for validating tracking quality or finding tracking errors.
percflylost	% of time the tracker lost an animal. This parameter is useful for validating tracking quality or finding tracking errors.
stripedeviationmedian	Applies to Buridan data. Medain angular distance to the closest (in terms of azimuthal rotation) stripe when walking in the center of the arena in °.
turnsnumber	Applies to Y -maze data. Number of turns. One turn is defined as an animal (measured at center point) walking from one Y-maze arm 3mm into another.
turnsbias	Ratio of right VS left turns. Values larger than 0.5 indicate right -handedness. Values smaller than 0.5 indicate left-handedness.
