# Supplementary material for: Individuality across environmental context in Drosophila melanogaster
Source: eLife. 2026 Apr 13;13:RP98171. doi: 10.7554/eLife.98171 (PMC13075937; doi:10.7554/eLife.98171)
Supplement: Supplementary file 2. [file elife-98171-supp2.pdf]

LED Buridan Setup

Nr	Description	Amount
1	Flir Firefly S FFY-U3-04S2M-C + Edmund optics UC Series Fixed Focal Length Objective (8mm, Stock #33-307)	1
2	Acrylic plate white opaque matt 500 x 410 x 3 mm (30mm circular hole in center)	1
3	Clear acrylic cylinder (300mm diameter, 300mm height) + diffuser paper or satinice acrylic cylinder (300mm diameter, 300mm height)	1
4	Fluorscent tubes (Osram, L 40w, 640C circular cool white) + Osram QT-M 1626–42 controller for AC flicker rate >1kHz	4
5	Aluminium profiles black (45 x 45 mm) P45N10LS www.aluprofile24.de:	
	400mm + thread M12 (min 100mm) profile	4
	480mm profile	2
	485mm profile	1
	+ Construction accessories www.aluprofile24.de:	
	Wi45x45 angle	10
	IWiN10 angle	20
	BuMuM8 nut screw	20
	FußD40DG m12x100 feet	4
	+ BitFenix Spectre PRO 230 mm mounted between aluminium rails on 2 opposing sides of the setup for cooling the LED matrix	8
6	6x RGB LED matrix (128 x 64 pixel) www.ledall.de 1x Hdmi controller Novastar mctrl 660 pro + 1x Novastar A8S receiving card 6x	1
	+ Famotec locating ring 3mm inner diameter (EAN: 4053199955619) + grub screw M3 x 20mm for fixing LED matrix to the LED cylinder	150
7	Transparent acrylic dome 120mm diameter (www.architekturbedarf.de) coated with Sigmacote on the inside	1
8	PLEXIGLAS® WH10 DC white snow satinice disk 119 x 119 x 10 mm + 850nm LED strip wrapped around edge for backlight illumintaion	1
9	Acrylic plate white opaque matt 500 x 500 x 3 mm (120mm circular hole in center)	1
10	PLEXIGLAS® WH10 DC white snow satinice 500 x 500 x 3 mm	1
11	PLEXIGLAS® WH10 DC white snow satinice disk 119 x 119 x 10 mm + 850nm LED strip (www.ledxess.de) wrapped around edge for backlight illumintaion	1
12	PLEXIGLAS® WH10 DC white snow satinice 500 x 500 x 10 mm (300mm circular hole in center)	2

IndyTrax Setup

Nr	Description	Amount
13	120x12mm EEC0381B1-000U-A99 Sunon	4
14	880nm infrared backlight https://www.amazon.de/LED-Lichttherapie-Dunkelrotes-Infrarot-LED-Lichttherapie-Panel-Hautverj%C3%BCngung-Druckentlastung/dp/B07ZQ	1
15	Server cabinet https://www.serverschrank24.de/15-he-serverschrank-wandgehause-mit-glastur-bxtxh-600-x-450-x-820-mm.html	1
16	Translucent acrylic cylinder https://www.kus-kunststofftechnik.de/plexiglas-rohr/plexiglasxt-satiniert-o-60-mm-laenge-waehlbar-von-100-bis-1400-mm/	25
16	Electroluminescent foil A7 white	50
16	Transparent dome 50mm https://www.dekoweltcreativ.de/acrylkugel-transparent-teilbar-stueck/p-3330.html	25
16	Plexiglas GS white 70% 3mm WH10 49mm diameter https://www.plattenzuschnitt24.de/Plexiglas-GS-weiss-WH10-GT-3mm.html	50
17	19" drawer https://www.it-budget.de/19-Fachboden-ausziehbar-Vollauszug-Tragkraft-30-kg-350-mm-schwarz	2
18	Camera Point Grey BFLY-PGE-23S6C-C mounted on Rack chassis 24563-194nVent – Schroff	1
18	Objective LM8HC3HOLE 1" 8mm/F1.4	1
19	BitFenix Spectre PRO 230 mm	2
20	Carbon heater https://thermowelt.de/Artikel/carbonheater1-1GN-220V.html	1
**Controllers**	Humidity controller TXG top-messtechnik.com TMT-HC-210 controlls 40x40mm Sunon EE40101S1-1000U-999 Axiallüfter 12 V/DC placed on (4)	1
	PID temperature controller pohltechnic.com RT4-121-Tr21Sd controlls (20)	1
	VOLTCRAFT LSP-1165 for controlling IR LED panel (14) and fans (18, 19)	2
	TT COMMANDER FT for cooling down enclosure, if necessary (controlls enclosure fans (13))	1
	Inverter https://www.el-light.shop/epages/17778431.sf/de_DE/?ObjectPath=/Shops/17778431/Products/InF1S150 controlls el-foil (16) brightness	1

LED flight simulator

Nr	Description	Amount
21	Aluminium profiles black (30 x 30 mm) P30N8 www.aluprofile24.de:	
	600mm profile	4
	165mm profile	10
	200mm profile	2
	+ Construction accessories www.aluprofile24.de:	
	PADK30N8B cap	4
	IWIN8B angle	25
	BuMuM6 nut screw	10
	WI30N8B	10
	HaKoM6 (12mm)	20
22	optional servo motor for dorsal stimulus presentation (Dynamixel MX-12W) + ball bearing 6811-ZZ (www.kugellager-express.de)	1
23	4x RGB LED matrix (128 x 64 pixel) www.ledall.de + 1x Hdmi controller Novastar mctrl 660 pro + 1x Novastar A8S receiving card	1
	+ Famotec locating ring 3mm inner diameter (EAN: 4053199955619) + grub screw M3 x 20mm for fixing LED matrix to the LED cylinder	150
24	Neodynium cylinder magnet (5mm diameter, 20mm height) + white plastic cap + 1mm diameter v-shaped sapphire bearing in center for friction reduction	1
25	Neodynium ring magnet (10mm diameter, 5mm height, inner diameter 5mm) + white plastic cap	1
26	850nm near infrared LED array (https://www.amazon.de/POFET-Infrarot-IR-LED-850-nm-Platinenmodul-f%C3%BCr-das-Kamera%C3%Bcberwachungssystem)	1
27	Flir Firefly S FFY-U3-04S2M-C + Edmund optics UC Series Fixed Focal Length Objective (12mm, Stock #33-307)	1
**Air puffs**	Arduino UNO Rev3 + relais module + Schego membrane pump 300l/h	1
