# Supplementary material for: Individuality across environmental context in Drosophila melanogaster
Source: eLife. 2026 Apr 13;13:RP98171. doi: 10.7554/eLife.98171 (PMC13075937; doi:10.7554/eLife.98171)
Supplement: Supplementary file 3. — For each behavioral trait, the model included environmental context variables as fixed effects and fly identity (ID) as a random intercept. Name: fixed-effect terms. Estimate: estimated effect size on the respective trait (in trait units). SE: standard error of estimate. DF: degrees of freedom used for testing significance. tStat: t-statistic. p-value: p-value. Lower/upper: bound of the 95% confidence interval for the effect estimate. Trait: behavioral trait. [file elife-98171-supp3.pdf]

Walking								
**Name**	**Estimate**	**SE**	**tStat**	**DF**	**pValue**	**Lower**	**Upper**	**Trait**
**(Intercept)**	-36.0989	1.703036	-21.1968	2977	5.45E-93	-39.4381	-32.7596	% of time walked
**Day**	0.114762	0.525089	0.218558	2977	0.827009	-0.91481	1.144337	% of time walked
**Temp_C**	1.641446	0.045068	36.4213	2977	1.57E-240	1.553078	1.729814	% of time walked
**StripeNum**	2.287736	0.401177	5.702555	2977	1.30E-08	1.501123	3.074349	% of time walked
**Illumination**	4.202395	0.644612	6.519264	2977	8.27E-11	2.938465	5.466325	% of time walked
**ArenaType**	-0.18729	0.782171	-0.23945	2977	0.81077	-1.72094	1.346356	% of time walked
**(Intercept)**	8.535013	0.29851	28.59203	2906	1.19E-158	7.9497	9.120327	Walking speed (in mm/s)
**Day**	0.708806	0.085192	8.32006	2906	1.33E-16	0.541762	0.87585	Walking speed (in mm/s)
**Temp_C**	0.228039	0.008173	27.89993	2906	5.54E-152	0.212013	0.244066	Walking speed (in mm/s)
**StripeNum**	0.582187	0.071393	8.154717	2906	5.15E-16	0.442202	0.722173	Walking speed (in mm/s)
**Illumination**	0.57637	0.114911	5.015818	2906	5.60E-07	0.351056	0.801685	Walking speed (in mm/s)
**ArenaType**	-3.52742	0.130363	-27.0584	2906	5.31E-144	-3.78303	-3.2718	Walking speed (in mm/s)
**(Intercept)**	0.740154	0.033723	21.94792	2660	2.87E-98	0.674028	0.80628	Vector strength
**Day**	0.039671	0.008519	4.656816	2660	3.37E-06	0.022967	0.056375	Vector strength
**Temp_C**	-0.02178	0.000899	-24.2139	2660	3.19E-117	-0.02354	-0.02002	Vector strength
**StripeNum**	0.115137	0.007552	15.24591	2660	2.22E-50	0.100329	0.129946	Vector strength
**Illumination**	-0.04424	0.011957	-3.70031	2660	0.00022	-0.06769	-0.0208	Vector strength
**ArenaType**	0.000995	0.013366	0.074413	2660	0.940687	-0.02521	0.027204	Vector strength
**(Intercept)**	6.076388	4.194318	1.448719	2093	0.147566	-2.14908	14.30186	Angularvelocity (in °/s)
**Day**	0.724146	0.969316	0.747069	2093	0.455106	-1.17678	2.625069	Angularvelocity (in °/s)
**Temp_C**	-0.14832	0.121757	-1.21813	2093	0.223311	-0.38709	0.090461	Angularvelocity (in °/s)
**StripeNum**	0.153049	0.930464	0.164487	2093	0.869364	-1.67168	1.97778	Angularvelocity (in °/s)
**Illumination**	-1.77591	1.572772	-1.12916	2093	0.258959	-4.86027	1.308446	Angularvelocity (in °/s)
**ArenaType**	0.471883	1.721123	0.274171	2093	0.78398	-2.90341	3.847173	Angularvelocity (in °/s)
**(Intercept)**	1.016901	0.038389	26.48924	2981	4.94E-139	0.941629	1.092173	Centrophobicity
**Day**	-0.02827	0.009991	-2.82914	2981	0.004699	-0.04786	-0.00868	Centrophobicity
**Temp_C**	0.0064	0.001061	6.031474	2981	1.82E-09	0.00432	0.008481	Centrophobicity
**StripeNum**	0.023118	0.008831	2.617863	2981	0.008893	0.005803	0.040433	Centrophobicity
**Illumination**	-0.05418	0.014117	-3.83793	2981	0.000127	-0.08186	-0.0265	Centrophobicity
**ArenaType**	-0.33056	0.015574	-21.2253	2981	3.16E-93	-0.3611	-0.30002	Centrophobicity

Flight								
**Name**	**Estimate**	**SE**	**tStat**	**DF**	**pValue**	**Lower**	**Upper**	**Trait**
**(Intercept)**	1.405556	1.082089	1.298927	177	0.195658	-0.7299	3.541013	Number of pauses
**Day**	-0.05556	0.519965	-0.10684	177	0.915033	-1.08168	0.970573	Number of pauses
**Contrast**	0.725	0.318412	2.276924	177	0.023988	0.096627	1.353373	Number of pauses
**(Intercept)**	585.0846	125.6295	4.657222	177	6.27E-06	337.1601	833.0091	Abs ang velocity sum (in °/min)
**Day**	5.265697	57.14176	0.092151	177	0.926682	-107.501	118.0325	Abs ang velocity sum (in °/min)
**Contrast**	61.74883	34.99204	1.764654	177	0.079347	-7.30646	130.8041	Abs ang velocity sum (in °/min)
**(Intercept)**	0.9028	0.019721	45.77796	177	4.87E-100	0.863881	0.94172	Vector strength
**Day**	-0.01242	0.009861	-1.25997	177	0.209338	-0.03188	0.007035	Vector strength
**Contrast**	0.021463	0.006038	3.554403	177	0.000486	0.009546	0.033379	Vector strength
**(Intercept)**	0.703611	0.741426	0.948997	177	0.343916	-0.75956	2.166784	Angular velocity (in °/s)
**Day**	-0.51914	0.329735	-1.57442	177	0.117176	-1.16986	0.131577	Angular velocity (in °/s)
**Contrast**	-0.07577	0.20192	-0.37525	177	0.70792	-0.47425	0.32271	Angular velocity (in °/s)
**(Intercept)**	218.6298	8.189625	26.69595	177	5.80E-64	202.468	234.7917	Heading (in °)
**Day**	-4.84511	4.094813	-1.18323	177	0.238304	-12.926	3.235827	Heading (in °)
**Contrast**	-44.6257	2.50755	-17.7965	177	2.79E-41	-49.5742	-39.6772	Heading (in °)
