# Supplementary material for: Individuality across environmental context in Drosophila melanogaster
Source: eLife. 2026 Apr 13;13:RP98171. doi: 10.7554/eLife.98171 (PMC13075937; doi:10.7554/eLife.98171)
Supplement: MDAR checklist [file elife-98171-mdarchecklist1.pdf]

Materials Design Analysis Reporting (MDAR) Checklist for Authors

The MDAR framework establishes a minimum set of requirements in transparent reporting mainly applicable to studies in the life sciences.

eLife asks authors to **provide detailed information within their article** to facilitate the interpretation and replication of their work. Authors can also upload supporting materials to comply with relevant reporting guidelines for health-related research (see EQUATOR Network), life science research (see the BioSharing Information Resource), or animal research (see the ARRIVE Guidelines and the STRANGE Framework; for details, see *eLife*'s Journal Policies). Where applicable, authors should refer to any relevant reporting standards materials in this form.

For all that apply, please note **where in the article** the information is provided. Please note that we also collect information about data availability and ethics in the submission form.

Materials:

Newly created materials	Indicate where provided: section/figure legend	N/A
The manuscript includes a dedicated "materials availability statement" providing transparent disclosure about availability of newly created materials including details on how materials can be accessed and describing any restrictions on access.	The manuscript describes newly created behavioral hardware (IndyTrax assay modules, 3D-printable arena components) and custom analysis code. 3D-printable parts, assay files, and analysis code are available via the project GitHub repository github.com/LinneweberLab/Mathejczyk_2024_eLife_Individuality; a parts list is provided in Supplementary File 2.	

Antibodies	Indicate where provided: section/figure legend	N/A
For commercial reagents, provide supplier name, catalogue number and RRID, if available.		N/A. No antibodies were used in this study.

DNA and RNA sequences	Indicate where provided: section/figure legend	N/A
Short novel DNA or RNA including primers, probes: Sequences should be included or deposited in a public repository.		N/A. No DNA or RNA reagents were generated or used in this study.

**Cell materials**	**Indicate where provided: section/figure legend**	**N/A**
Cell lines: Provide species information, strain. Provide accession number in repository OR supplier name, catalog number, clone number, OR RRID.		N/A. No cell lines or primary cell cultures were used in this study.
Primary cultures: Provide species, strain, sex of origin, genetic modification status.		N/A. No cell lines or primary cell cultures were used in this study.

**Experimental animals**	**Indicate where provided: section/figure legend**	**N/A**
Laboratory animals or Model organisms: Provide species, strain, sex, age, genetic modification status. Provide accession number in repository OR supplier name, catalog number, clone number, OR RRID.	As stated in the Materials and methods: One-week-old wild-type *Drosophila melanogaster* were used. The used strains were CantonS and TopBanana. Flies were reared on standard yeast-cornmeal medium at 25°C and 50% relative humidity under a 12 h:12 h light:dark cycle. Sex-specific analyses are reported in multiple figure supplements; females and males were analyzed separately.	
Animal observed in or captured from the field: Provide species, sex, and age where possible.		N/A. No wild caught animals were used in this study.

**Plants and microbes**	**Indicate where provided: section/figure legend**	**N/A**
Plants: provide species and strain, ecotype and cultivar where relevant, unique accession number if available, and source (including location for collected wild specimens).		N/A. No plants were used in this study
Microbes: provide species and strain, unique accession number if available, and source.		N/A. No microbes were used in this study

**Human research participants**	**Indicate where provided: section/figure legend) or state if these demographics were not collected**	**N/A**

If collected and within the bounds of privacy constraints report on age, sex, gender and ethnicity for all study participants.		N/A. This study did not involve human participants.

Design:

**Study protocol**	**Indicate where provided: section/figure legend**	**N/A**
If the study protocol has been pre-registered, provide DOI. For clinical trials, provide the trial registration number OR cite DOI.		N/A. The study was not preregistered.

**Laboratory protocol**	**Indicate where provided: section/figure legend**	**N/A**
Provide DOI OR other citation details if detailed step-by-step protocols are available.	Detailed assay construction, tracking, and analysis procedures are described in the Materials and methods and associated figure supplements. Step-by-step setup files and code are available via the project GitHub repository. github.com/LinneweberLab/Mathejczyk_2024_eLife_Individuality	

**Experimental study design (statistics details) ***		
**For in vivo studies: State whether and how the following have been done**	**Indicate where provided: section/figure legend. If it could have been done, but was not, write "not done"**	**N/A**
Sample size determination	Sample sizes are reported in the Materials and methods and in figure legends. No formal power analysis was performed. Sample sizes were chosen based on assay throughput, repeated-measures design, and consistency with prior work using these behavioral paradigms	
Randomisation	Not done. Flies were not assigned to experimental conditions using formal randomization.	

Blinding	Not done. No formal blinding was used during data collection or analysis. Behavioral tracking and quantification were automated using custom-written software.	
Inclusion/exclusion criteria	No animals or data points were excluded from analysis except in cases where recordings failed, tracking quality was insufficient for reliable quantification or behavioral parameters could not be extracted because the animal did not move during the recording. No additional attrition criteria were pre-established.	

**Sample definition and in-laboratory replication**	**Indicate where provided: section/figure legend**	**N/A**
State number of times the experiment was replicated in the laboratory.	The experimental unit was an individual fly. Data represent biological replicates (individual animals), not technical replicates. Repeated measurements of the same fly across contexts/days were used in within-animal comparisons. Sample sizes for each assay are given in the Materials and methods, and figure legends report animal numbers for individual experiments.	
Define whether data describe technical or biological replicates.	Data represent biological replicates as provided by the figure legends.	

**Ethics**	**Indicate where provided: section/submission form**	**N/A**
Studies involving human participants: State details of authority granting ethics approval (IRB or equivalent committee(s), provide reference number for approval.		N/A. This study did not involve human participants.

Studies involving experimental animals: State details of authority granting ethics approval (IRB or equivalent committee(s), provide reference number for approval.		N/A. No specific animal ethics approval is required to work with *Drosophila melanogaster* under institutional and national regulations. Flies were maintained and tested under standard laboratory conditions
Studies involving specimen and field samples: State if relevant permits obtained, provide details of authority approving study; if none were required, explain why.		N/A. No field collections or specimen sampling were performed.

**Dual Use Research of Concern (DURC)**	**Indicate where provided: section/submission form**	**N/A**
If study is subject to dual use research of concern regulations, state the authority granting approval and reference number for the regulatory approval.		N/A. This study is not subject to dual use research of concern regulations.

Analysis:

**Attrition**	**Indicate where provided: section/figure legend**	**N/A**
Describe whether exclusion criteria were pre-established. Report if sample or data points were omitted from analysis. If yes, report if this was due to attrition or intentional exclusion and provide justification.	No animals or data points were excluded from analysis except in cases where recordings failed or tracking quality was insufficient for reliable quantification. No additional attrition criteria were pre-established	

**Statistics**	**Indicate where provided: section/figure legend**	**N/A**
Describe statistical tests used and justify choice of tests.	Statistical analyses included correlation analyses, generalized linear modelling, ridge-regression-based rank-shift analyses, and hierarchical linear mixed-effects modelling, as described in the Results and Materials and methods. Fixed-effect summaries from hierarchical linear mixed-model analyses are provided in Supplementary File 3.	

	Exact sample sizes are reported in the Materials and methods and figure legends.	

**Data availability**	**Indicate where provided: section/submission form**	**N/A**
For newly created and reused datasets, the manuscript includes a data availability statement that provides details for access (or notes restrictions on access).	Available in the Data availability statement in the Materials and methods. All experimental data are available here:https://doi.org/10.5281/zenodo.18342691.	
When newly created datasets are publicly available, provide accession number in repository OR DOI and licensing details where available.	Available in the Data availability statement in the Materials and methods. All experimental data are available here:https://doi.org/10.5281/zenodo.18342691.	
If reused data is publicly available provide accession number in repository OR DOI, OR URL, OR citation.		N/A

**Code availability**	**Indicate where provided: section/figure legend**	**N/A**
For any computer code/software/mathematical algorithms essential for replicating the main findings of the study, whether newly generated or re-used, the manuscript includes a data availability statement that provides details for access or notes restrictions.	Available in the Data availability statement in the Materials and methods. The code for the analyses presented in this paper is openly accessible atgithub.com/LinneweberLab/Mathejczyk_2024_eLife_Individuality	
Where newly generated code is publicly available, provide accession number in repository, OR DOI OR URL and licensing details where available. State any restrictions on code availability or accessibility.	Available in the Data availability statement in the Materials and methods. The code for the analyses presented in this paper is openly accessible atgithub.com/LinneweberLab/Mathejczyk_2024_eLife_Individuality	
If reused code is publicly available provide accession number in repository OR DOI OR URL, OR citation.		N/A

Reporting:

The MDAR framework recommends adoption of discipline-specific guidelines, established and endorsed through community initiatives.

Adherence to community standards	Indicate where provided: section/figure legend	N/A
State if relevant guidelines (e.g., ICMJE, MIBBI, ARRIVE, STRANGE) have been followed, and whether a checklist (e.g., CONSORT, PRISMA, ARRIVE) is provided with the manuscript.	Reporting was guided by the eLife MDAR framework. The study also follows transparency principles relevant to animal-behavior research, including clear reporting of biological replicates, sample sizes, assay conditions, and code/material availability	

---

* We provide the following guidance regarding transparent reporting and statistics; we also refer authors to [Ten common statistical mistakes to watch out for when writing or reviewing a manuscript].

Sample-size estimation

- You should state whether an appropriate sample size was computed when the study was being designed
- You should state the statistical method of sample size computation and any required assumptions
- If no explicit power analysis was used, you should describe how you decided what sample (replicate) size (number) to use

Replicates

- You should report how often each experiment was performed
- You should include a definition of biological versus technical replication
- The data obtained should be provided and sufficient information should be provided to indicate the number of independent biological and/or technical replicates
- If you encountered any outliers, you should describe how these were handled
- Criteria for exclusion/inclusion of data should be clearly stated
- High-throughput sequence data should be uploaded before submission, with a private link for reviewers provided (these are available from both GEO and ArrayExpress)

Statistical reporting

- Statistical analysis methods should be described and justified
- Raw data should be presented in figures whenever informative to do so (typically when N per group is less than 10)
- For each experiment, you should identify the statistical tests used, exact values of N, definitions of center, methods of multiple test correction, and dispersion and precision measures (e.g., mean, median, SD, SEM, confidence intervals; and, for the major substantive results, a measure of effect size (e.g., Pearson's r, Cohen's d)
- Report exact p-values wherever possible alongside the summary statistics and 95% confidence intervals. These should be reported for all key questions and not only when the p-value is less than 0.05.

Group allocation

- Indicate how samples were allocated into experimental groups (in the case of clinical studies, please specify allocation to treatment method); if randomization was used, please also state if restricted randomization was applied
- Indicate if masking was used during group allocation, data collection and/or data analysis
